# Pseudobullous pilomatricoma: A rare variant of pilomatricoma

**DOI:** 10.1002/ski2.115

**Published:** 2022-04-02

**Authors:** Tung‐Chun Lee, Yan Liu, Ya‐Mei Zhang, Yike Huang, Zhi‐Yan Wang, Gui‐Qing Lu

**Affiliations:** ^1^ Department of Dermatology BenQ Medical Center The Affiliated BenQ Hospital of Nanjing Medical University Nanjing China; ^2^ Department of Dermatology Xiamen Chang Gung Hospital Xiamen China

## Abstract

Pilomatricoma (PM; calcifying epithelioma of Malherbe) is an uncommon adnexal tumour originating from the matrix of the hair follicles. Bullous appearance is a rare variant of PM, and its pathogenesis remains unclear. Here, we present a case of a 17‐year‐old girl with a pseudobullous PM on the right shoulder. Lymphatic dilatation and collagen disorder were histopathologically observed in this case, which may provide clues to elucidate the pathogenesis of pseudobullous PM.

## Graphical abstract

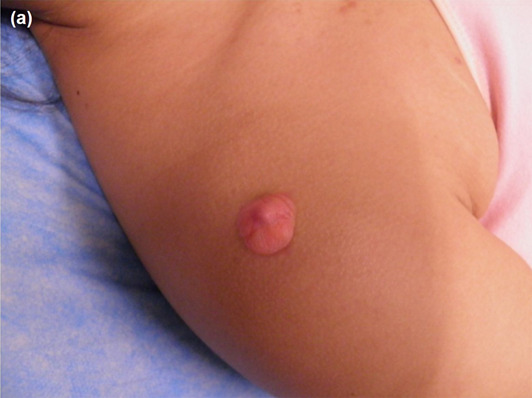

1

**What is already known about this topic?**

- The pseudobullous PM is a rare clinical type of PM; this particular type of PM most commonly affects the arm and shoulder.

**What does this study add?**

- Our report could enrich the diagnosis of bullous‐like disorders and provide clues to elucidate the pathogenesis of pseudobullous pilomatricoma.

## 2 INTRODUCTION

Pilomatricoma (PM; calcifying epithelioma of Malherbe) is an uncommon adnexal tumour originating from the matrix of the hair follicles with mutations in genes encoding β‐catenin (encoded by *CTNNB1*).^1^ Herein, we present an unusual case of pseudobullous PM on the right shoulder and report its clinical and histopathologic findings.

## 3 CASE REPORT

**FIGURE 1:**
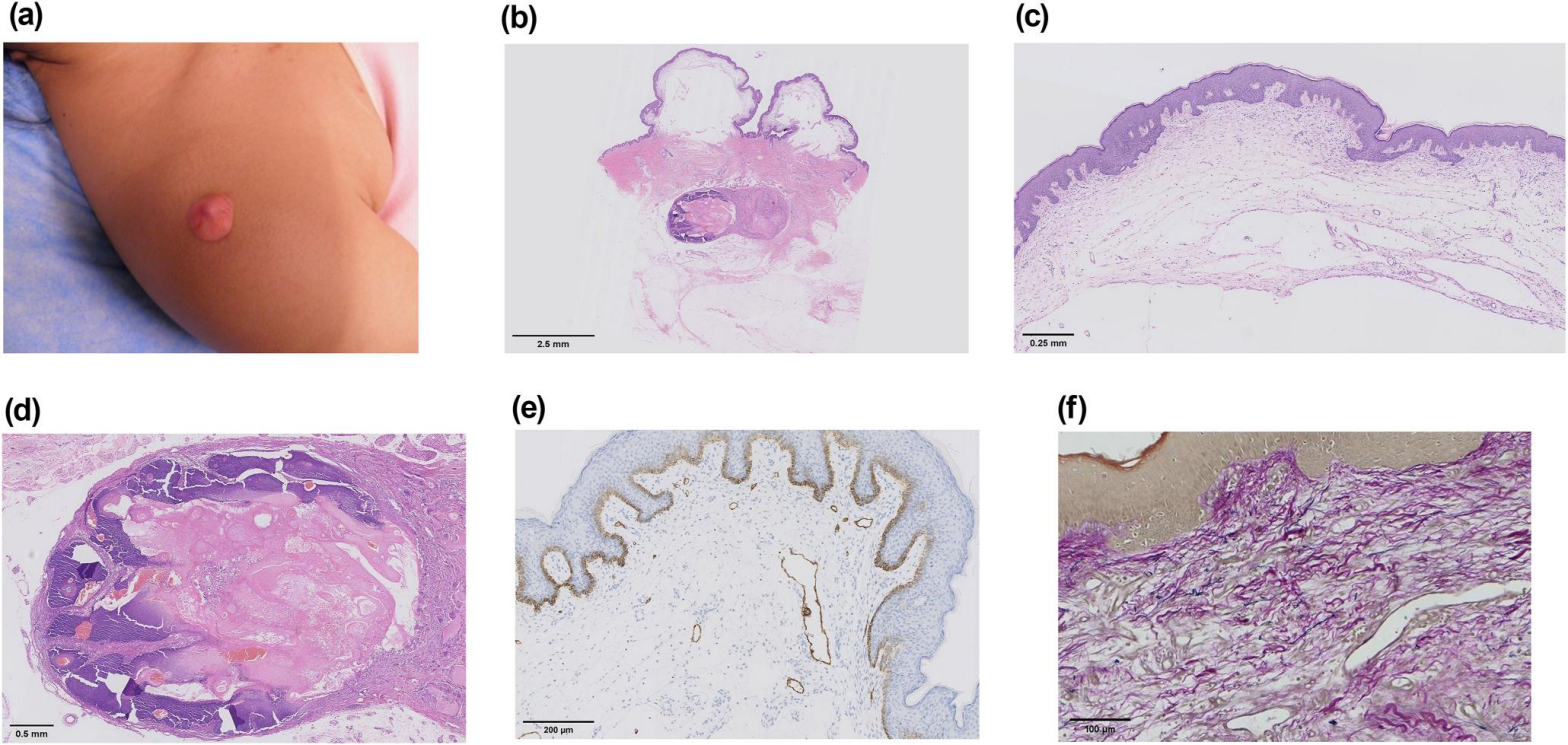
(a) A single, pink‐colored bullous‐like lesion on the right shoulder. Histopathological findings of the excised tissue; (b) histopathology showed oedema in the superficial dermis, tumour nests with fibrous capsule in the deep dermis. (H&E × 0.45); (c) dilated thin‐walled vascular structures within the upper dermis, representing dilated lymphatics, with marked oedema leading to disruption and separation of collagen bundles. (H&E × 5.0); (d) deeper in the dermis, there was an admixture of basaloid and ghost cells with focal calcifications, consistent with pilomatricoma. (H&E × 2.5); (e) lymphatic vessels were staining positive for D2‐40 in the upper dermis. (D2‐40 × 10); (f) near total loss of elastic fibres, and mild collagen disruption. (Verhoeff–Van Gieson × 20)

A 17‐year‐old girl presented a 6‐month solitary, asymptomatic, pink‐colored nodule on the right shoulder. The skin over the nodule had transformed into a bulla 5 months earlier. There was no particular local trauma, scratch, or injection history. Her past medical, household and societal accounts were quite unexceptional. Physical examination revealed a 2.0 × 2.0 cm pink bullous‐like lesion with an underlying hard nodule on the right shoulder (Figure 1a). Haematoxylin‐eosin staining revealed dilated, thin‐walled vascular structures within the upper dermis, representing dilated lymphatics, with marked oedema leading to disruption and separation of collagen bundles (Figure 1b,c). Deeper in the dermis, there were nests of tumours composed of basophilic and shadow cells (ghost cells), with focal calcifications surrounded by a fibrous capsule (Figure 1d). The immunohistochemical results showed positive vascular staining for D2‐40 in the superficial dermis and marked dilation of the lymphatic vessels (Figure 1e). Verhoeff–Van Gieson staining revealed near‐total loss of elastic fibres and mild collagen disruption (Figure 1f). Based on these findings, she was diagnosed with pseudobullous PM. The neoplasm was surgically removed. Presently, the patient is on follow‐up, and there has been no post‐surgical recurrence.

## 4 DISCUSSION

PM usually occurs as a solitary, painless, and slow‐growing tumour on the head and neck. Multinodular, familial, exophytic, bullous, anetodermic, perforating, and giant pilomatricomas have been reported in the literature.^2^ The incidence of PM with a bullous appearance is between 3% and 6%.^3^ This particular type of PM most commonly affects females aged 10–20 years, in the arm and shoulder.^4^ Although PM can be associated with genetic disorders, such as myotonic dystrophy and Turner's syndrome, pseudobullous PM is unrelated to genetic disorders as per the literature review by Chen et al.^5^ On histological examination, pseudobullous PM often presents with tumour nests in the deep dermis composed of basophilic and shadow cells (ghost cells), numerous dilated lymphatic vessels, and lymphoedema in the superficial dermis. The usual differential diagnosis for such lesions includes lymphangioma, malignant tumours, and bullous morphea.^4^, ^5^

Presently, the etiopathogenesis of pseudobullous PM is not clearly understood. Some reports suggest that the elastinolytic enzymes produced by tumour cells and lytic products may cause the decline of elastic fibres and demolition and dilation of the lymphatic vessels, causing lymph fluid accumulation in the dermis, and consequently forming a bullous appearance.^6^ However, the more widely accepted hypothesis is that the tumour nodule growth causes hindrance in the lymphatic vessels and congestion of lymphatic fluid. It subsequently induces the dilation of lymphatic vessels and subsequent leakage of lymphatic fluid, producing a bullous appearance.^3^ ^,^ ^6^ In our case, both lymphatic dilatation and lack of collagen fibres could be demonstrated. Therefore, we speculated that compression of the lymphatic vessels and long‐term mechanical irritation by the tumour might contribute to the occurrence of the bulla. Sufficient depth of surgical excision is the treatment of choice. Our report could enrich the diagnosis of bullous‐like disorders and provide clues to elucidate the pathogenesis of pseudobullous PM.

## CONFLICT OF INTEREST

The authors have no conflict of interest to declare.

## ETHICS STATEMENT

The study followed the guidelines of the Helsinki Declaration, and informed consent was obtained from the patient.

## AUTHOR CONTRIBUTIONS

**Tung‐Chun Lee:** Writing – original draft (lead). **Yan Liu:** Supervision (lead). **Ya‐Mei Zhang:** Software (lead). **Yike Huang:** Supervision (supporting). **Zhi‐Yan Wang:** Software (supporting). **Gui‐Qing Lu:** Writing – review & editing (lead).

## Data Availability

Data sharing is not applicable to this article as no new data were created or analyzed in this study.
